# A comparison of customised and prefabricated insoles to reduce risk factors for neuropathic diabetic foot ulceration: a participant-blinded randomised controlled trial

**DOI:** 10.1186/1757-1146-5-31

**Published:** 2012-12-05

**Authors:** Joanne S Paton, Elizabeth A Stenhouse, Graham Bruce, Daniel Zahra, Ray B Jones

**Affiliations:** 1 Faculty of Health, Education and Society, Plymouth University, Plymouth, UK; 2 Plymouth Community Health Care (CIC), Plymouth, UK; 3 Faculty of Science and Technology, Plymouth University, Plymouth, UK

## Abstract

**Background:**

Neuropathic diabetic foot ulceration may be prevented if the mechanical stress transmitted to the plantar tissues is reduced. Insole therapy is one practical method commonly used to reduce plantar loads and ulceration risk. The type of insole best suited to achieve this is unknown. This trial compared custom-made functional insoles with prefabricated insoles to reduce risk factors for ulceration of neuropathic diabetic feet.

**Method:**

A participant-blinded randomised controlled trial recruited 119 neuropathic participants with diabetes who were randomly allocated to custom-made functional or prefabricated insoles. Data were collected at issue and six month follow-up using the F-scan in-shoe pressure measurement system. Primary outcomes were: peak pressure, forefoot pressure time integral, total contact area, forefoot rate of load, duration of load as a percentage of stance. Secondary outcomes were patient perceived foot health (Bristol Foot Score), quality of life (Audit of Diabetes Dependent Quality of Life). We also assessed cost of supply and fitting. Analysis was by intention-to-treat.

**Results:**

There were no differences between insoles in peak pressure, or three of the other four kinetic measures. The custom-made functional insole was slightly more effective than the prefabricated insole in reducing forefoot pressure time integral at issue (27% vs. 22%), remained more effective at six month follow-up (30% vs. 24%, p=0.001), but was more expensive (UK £656 vs. £554, p<0.001). Full compliance (minimum wear 7 hours a day 7 days per week) was reported by 40% of participants and 76% of participants reported a minimum wear of 5 hours a day 5 days per week. There was no difference in patient perception between insoles.

**Conclusion:**

The custom-made insoles are more expensive than prefabricated insoles evaluated in this trial and no better in reducing peak pressure. We recommend that where clinically appropriate, the more cost effective prefabricated insole should be considered for use by patients with diabetes and neuropathy.

**Trial registration:**

Clinical trials.gov (NCT00999635). Note: this trial was registered on completion.

## Background

Peak plantar pressure is linked to the formation of neuropathic foot ulcers [1,2]. Reducing plantar pressure forms one important element of the ulcer prevention strategy, particularly in neuropathic feet with reduced or absent protective sensation, where plantar loads and tissue stress are increased [3-6]. Yet there are no good quality randomised controlled trials investigating the efficacy of offloading interventions for pressure reduction and ulcer prevention amongst individuals with diabetes and neuropathy [4,7].

Traditionally used, custom-made moulded insoles may reduce peak pressure by maximising total plantar contact area [8], but this method of pressure relief may not fully compensate for the underlying biomechanical foot dysfunction accompanying diabetic neuropathy. Biomechanical foot dysfunction influences plantar load distribution and mechanical tissue stress [9,10]. Greater influence over plantar loads and tissue stress may result if a more functional approach to insole design was used to minimise the impact of altered foot biomechanics. Few studies have investigated the application of biomechanical principles to reduce plantar tissue stress under diabetic neuropathic feet.

The main hypothesis of this study was that custom-made functional insoles would reduce risk factors intrinsic to the foot, in particular peak pressure, for neuropathic ulcers in diabetic feet over six months compared to prefabricated moulded insoles. However, given the limitations of peak pressure described above, we examined changes in four other kinetic variables: total contact area, forefoot pressure time integral, rate of forefoot load, and duration of load as a percentage of stance. Two preliminary studies were undertaken to select and test the suitability of the five kinetic outcome measures for purpose [11,12]. We also assessed patient-perceived foot health, health-related quality of life and cost.

## Methods

### Ethics

Ethics approval was granted by the Cornwall and Plymouth Research Ethics Committee (REC number: 05/Q2103/150).

### Design and sample size

This participant-blinded randomised controlled trial compared custom-made functional insoles with prefabricated moulded insoles to reduce abnormal plantar loads and tissue stress, risk factors for neuropathic ulcers in diabetic feet, over six months. Sample size was based on change in peak pressure using values sourced from Barnett [13]; she found a difference between 346.4 kPa for a moulded insole and 296.4 kPa for an inlay (standard deviation 80.1 kPa). A sample size of 54 in each group provided 90% power at *p* = 0.05, for a two-tailed test on peak pressure. The sample size was increased by 10% to allow for patient dropout giving a total of n=119.

### Recruitment

From March 2006 to October 2007, 119 consecutive patients fulfilling the enrolment criteria, attending for podiatry treatment and providing informed consent, were recruited at two centres in South West England. Patients were eligible if they: had Type 1 or 2 diabetes; had insensate or diminished sensation, defined as a deficit noted with the 10g monofilament and 128Hz tuning fork, using the method previously described [14]; had palpable or biphasic pulses; had not had a lower limb vascular or neuropathic ulceration (for at least the past six months); scored Grade 0 on the Wagner classification for foot ulcer; were able to walk a minimum of 10 metres unaided; and were willing to comply with the requirements of the study. Patients were excluded if they presented with severe fixed midfoot or rearfoot deformity such as that associated with Charcot arthropathy, a history of major bone or joint surgery of the lower limb, or were unable to comprehend simple instructions and comply with the study protocol.

### Randomisation and blinding

Participants were randomised in equal proportions to custom-made or prefabricated insoles, in blocks of six, independently for the two recruiting centres. Randomisation allocation was prepared by Plymouth University Statistics Department prior to patient recruitment. The primary investigator was blind to the intervention allocation until treatment commenced. Treatment allocation was revealed to the primary investigator by an independent observer following biomechanical examination, the casting of the foot and prescribing of insole and footwear. The clinical trial design was only single blind to accommodate unavoidable ethical and practical concerns regarding potential adverse effect. Participants remained blind to the intervention group assignment. At discharge from the trial, participants were asked to guess their intervention group assignment, providing estimates of the degree to which the blind allocation was maintained.

### Insoles

Based on the results of pilot work previously described [15], the insoles were constructed from a blue, medium density, 3 mm full length moulded ethylene vinyl acetate (EVA) base with a 6 mm full-length grey Poron® top cover. Although the two insoles had similar appearance they differed in design and construction.

#### Custom-made functional insole

**Figure 1:**
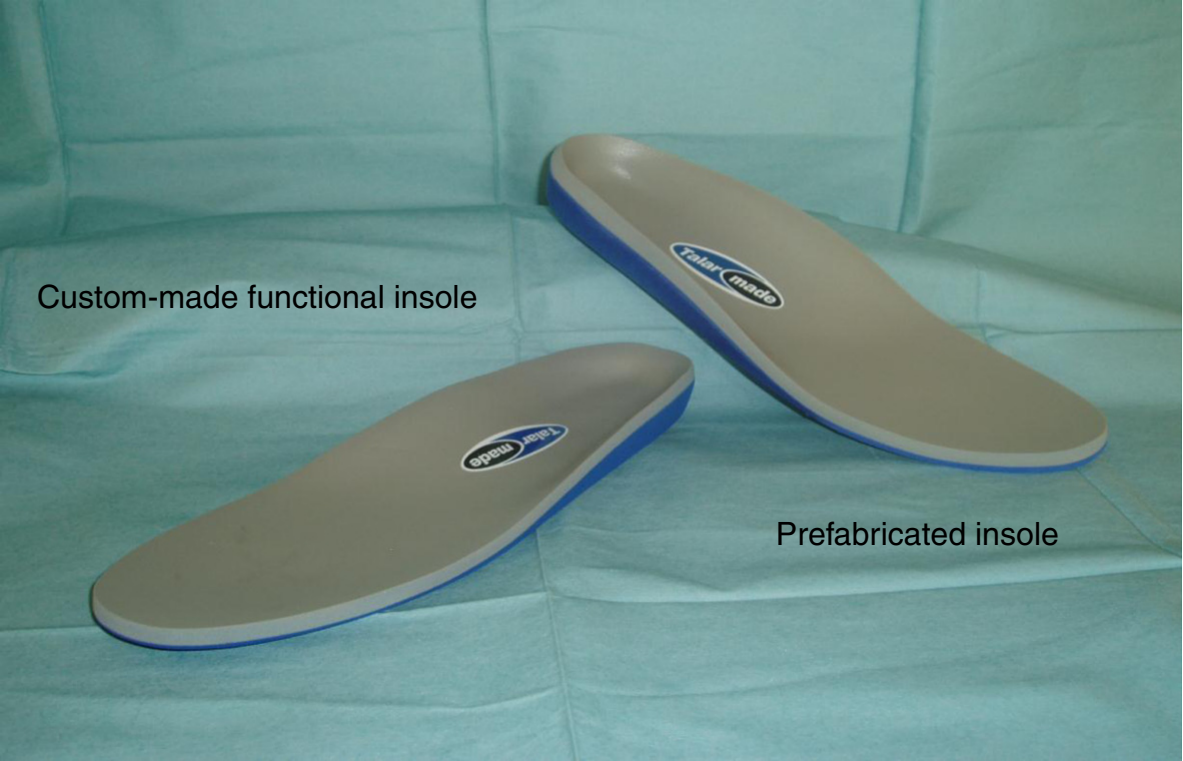
Example of the insoles used within the trial.

Each custom-made functional device was unique, fabricated from a cast of each individual foot, and prescribed and custom-manufactured to address individual biomechanical need in accordance with the prescription writing protocol. No detailed published prescription writing guidelines could be found for the treatment of this patient group. Therefore, a prescription writing protocol was devised for the trial based upon a critical analysis of the literature (See Additional file 1: Summary of prescription writing protocol and rational). A single experienced clinician conducted the lower limb biomechanical examination, obtained the foam box foot impression and wrote the insole prescription. The custom-made functional 3 mm EVA shells were milled from the cast using CAD/CAM technology (Figure 1).

#### Prefabricated insole

The prefabricated insole was ‘off the shelf’ and provided to fit the shoe. First Line Full Length Orthotics (Talarmade Ltd) consisted of a prefabricated full-length 3 mm medium EVA contoured shell covered in 6 mm Poron® (Figure 1). For the prefabricated insole group the biomechanical examination, casting and insole prescription writing process was undertaken using the same technique as for the custom-made functional group, but were not used.

#### Therapeutic footwear

Participants were also fitted with two pairs of modular non-bespoke therapeutic footwear self-selected from a choice of style and colour. The footwear incorporated the following specifications for patients with diabetes; softee leather upper, plain vamp, secure fastening, micro fibre lining material, padded collar, wall toe puff and a EVA micro rubber sole unit with rocker to forefoot positioned posterior to metatarsophlangeal joint line (County Orthopaedic Footwear Ltd).

### Primary outcome measures

Changes in kinetics were compared using the F-scan® in-shoe pressure analysis system (TEKSCAN® Ltd) between participants with custom-made functional and prefabricated insoles. Data were collected at two stages; insole issue and six months. The F-Scan® in-shoe pressure measurement system is capable of reliable and repeatable data collection [16,17]. To optimise the accuracy and repeatability of the data collected within this study the following precautions were incorporated within the data collection protocol. A new sensor was provided for each individual foot, labelled and used to collect data from that foot throughout the duration of the study. Prior to calibration each sensor underwent equilibration, was trimmed, and fitted into each shoe. Before each data collection session each patient was weighed and each pair of insoles calibrated against body weight. Following calibration, if sensor saturation pressure exceeded 2000 kPa the sensor was discarded. Calibration was then checked for within and between foot repeatability, and if excessive variation was observed, the sensor was re-calibration. Data were collected immediately following sensor calibration, recording two test conditions (with insoles and without insoles). Each test condition consisted of three walking trials, first a learning trial then two trials were recorded. The first and last step of each walking was excluded to allow for gait acceleration and deceleration. Gait velocity of each walking trial was timed to check for consistency, in the event variation was found the data was discarded and the test repeated. Participants were asked to walk between two chairs placed at either end of a walkway. Between the chairs, two marks were placed on the floor with a distance of ten metres between them. Using a stop watch, the time taken for the participant to walk the ten metre distance was recorded and then gait velocity was calculated in metres per second. Participants underwent debridement of plantar callus prior to data collection. Participants wore the standardised therapeutic footwear provided and 20 denier stockings during the collection of pressure data.

Data from one foot of each patient averaging a minimum of six steps was used within the analysis; selected as the foot recording the highest peak pressure value at baseline. Five kinetic outcome measures between the two groups, before and after the intervention, were used:

i. Peak pressure: highest value of peak pressure calculated by dividing force by total contact area.

ii. Total contact area: total loaded plantar surface area

iii. Forefoot pressure time integral: forefoot peak pressure multiplied by forefoot contact time, displayed as the area under the pressure/time curve. A forefoot mask was manually applied using the F-scan® software, saved as an object file and then downloaded onto subsequent trials to ensure consistency of size and position.

iv. Rate of forefoot loading: peak forefoot force of each foot strike divided by the time taken to reach peak force.

v. Duration of load at the site of highest peak pressure. Extracted using TAM analysis software (TEKSCAN® Ltd), the data is presented for the masked area as the duration and variation of load as a percentage of stance. The seven TAM analysis boxes were manually positioned to overlie seven anatomical regions of one movie display. The seven regions represented; the hallux, first metatarsal, second metatarsal, third and fourth metatarsal, fifth metatarsal, midfoot and heel. To ensure consistency, the box positions were saved to the system and then downloaded onto subsequent trials.

### Secondary outcome measures

Two self-report questionnaires validated for use with individuals diagnosed with diabetes generated the scores of perceived foot health (Bristol Foot Score) and health-related quality of life (Audit of Diabetes Dependent Quality of Life). The Bristol Foot Score is designed to measure an individual’s perception of foot health, including changes over time [18]. It contains 15 items grouped into 3 components; (i) concerns about feet and pain, (ii) footwear and general health, and (iii) mobility. The final score ranges from 15 representing the best possible perceived foot health to 73 corresponding with the worst. The Audit of Diabetes Dependent Quality of Life (ADDQoL) offers an individualised disease-specific health-related quality of life measure [19]. The ADDQoL allows participants to select domains of personal concern and rate them according to current quality and importance. The weighted domains are combined to produce an individualised summary score of health-related quality of life. The final score ranges from +9 representing a positive impact of diabetes on health-related quality of life to −9 representing a negative impact of diabetes on health-related quality of life.

### Cost

Costs of insole provision, including direct and indirect costs incurred in the supply and review of insoles and footwear were compared. The prefabricated and custom-made functional insoles cost £31.73 and £137.65 respectively. The provision of two pairs of therapeutic footwear per participant cost £300. Staff time was calculated using marginal costs based on the 2008 gross pay for a National Health Service (NHS) Band 7 clinician (UK) at an hourly rate of £53.51 inclusive of salary and oncosts. Participant travel and attendance costs were calculated from information provided from participant completed questionnaires at each clinic visit. Private transport costs were calculated on NHS rates for private car use (£0.53 per mile), whilst participant and accompanying person travel time was based upon the minimum wage for the United Kingdom (October 2008, £5.73 per hour).

### Adherence and adverse events

Other recorded data included patient adherence (monitored using a self-reported questionnaire completed at each clinic visit) and a record of adverse events.

### Statistical analysis

The data analysis was carried out using intention to treat including all participants randomised. Multiple imputations was used to address missing data, following the assumption that the data were missing at random such that the variables observed and recorded were predictive of the missing data. IBM SPSS statistics version 19 was used to impute the missing values and five imputed data sets were created.

All variables met assumptions for parametric data, therefore split plot ANOVAs using data collected at issue and six month follow-up were used to compare the custom-made functional and prefabricated insoles. Independent sample t-tests were used to compare the cost of providing the two different insoles. Statistical significance for hypothesis tests was set at 0.05.

## Results

### Participants and follow-up

**Figure 2:**
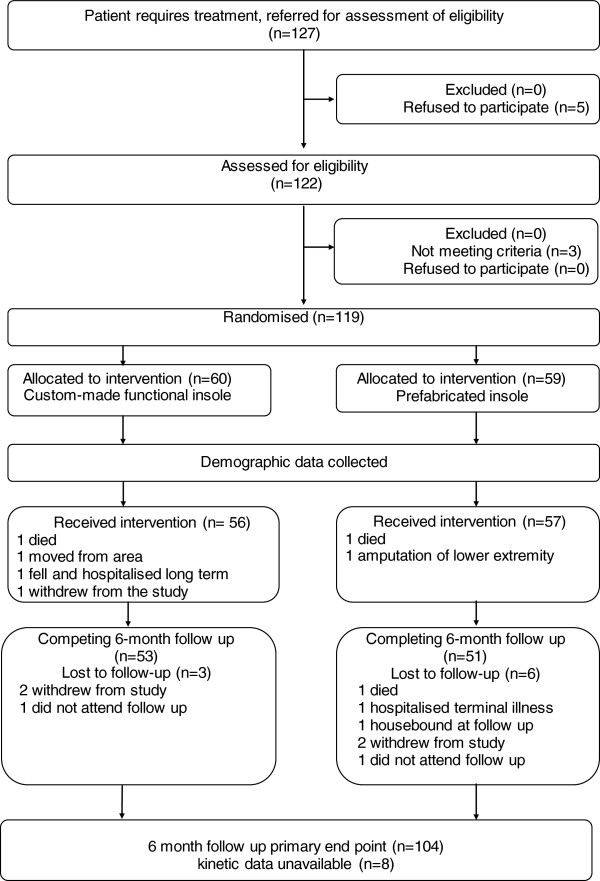
Participants journey through the trial.

A total of 127 people with diabetes and neuropathy requiring treatment were invited to be assessed for eligibility (Figure 2); five people declined and 122 were assessed. Three did not meet the entry criteria, leaving 119 who were recruited and randomised. Sixty participants were allocated the custom-made functional insole and 59 the prefabricated insole. Table 1 summarises the baseline characteristics of participants. A total of 104 (87%) participants completed the six month follow-up. The characteristics of participants failing to complete were similar between groups.

**Table 1:** Participant Characteristics

**Demographic Data**	**Custom**-**made group**	**Prefabricated group**
	(**n**=**60**)	(**n**=**59**)
Mean age in years (SD)	71 (10)	70 (10)
Mean weight in kg (SD)	95 (16)	97 (21)
Mean height in metres (SD)	1.74 (0.27)	1.69 (0.13)
Mean BMI (SD)	32 (10)	32 (11)
Gender male/female	48/12	42/17
Diabetes Type I/II/unknown	2/57/1	2/57/0
Neuropathy	60	59
PVD	0	0
HbA1c %	8.1 (2.3)	7.7 (2.9)
Mean duration of diabetes in years (SD)	9 (9)	9 (8)
Plantar callus (yes/no)	25/35	22/37
Toe deformity (yes/no)	30/29 (1 missing)	29/30
Prior foot ulcer (yes/no)	5/55	6/53
Mean left foot peak pressure in kPa (SD)	499 (137)	498 (135)
Mean right foot peak pressure in kPa (SD)	494 (142)	512 (174)
Mean forefoot PTI in kPa.sec (SD)	53 (17)	55 (16)
Mean rate of forefoot loading in kPa/sec (SD)	118 (35)	126 (41)
Mean total contact area in mm^2^ (SD)	14778 (1996)	14171 (2083)
Foot type (Foot Posture Index ≥4)	25	19

Note: PTI = Pressure time integral.

### Compliance in using the insoles

Of the 104 participants completing the six month follow-up, 41 (40%) reported full compliance, i.e. reported wearing insoles and shoes for a minimum of seven hours per day, seven days a week for a period of six months, whilst 79 (76%) reported 50% compliance equating to wearing the insoles and shoes for a minimum of five hours a day five days a week for the duration of the study.

### Insoles versus no-insoles

With the application of either type of insole there were significant reductions in peak pressure, forefoot pressure time integral and rate of forefoot loading, while total contact area increased compared to no insole in the shoe. For example, for the prefabricated insole, the peak pressure was reduced from 566 to 363 kPa, the pressure time integral from 57 to 45 kPa.sec, the reduction in forefoot rate of load from 126 to 119 kPa/sec, and the total contact area increased from 14219 to 18199 mm^2^.

### Comparison of insoles

There was no difference between custom-made functional and prefabricated insoles for peak pressure. Forefoot pressure time integral showed greater percent reduction for the custom made functional insole at issue (27% vs. 22%, *p*=0.001) and at six months (30% vs. 24%, *p*=0.001) (Table 2). The effect size was medium (Partial Eta Squared 0.89). There was no difference for total contact area, rate of forefoot loading, or duration of load. In addition to the plantar pressure data, there were no differences between custom-made functional insoles and prefabricated insoles in participants’ Bristol Foot Score or Audit of Diabetes Dependent Quality of Life (Table 3). The custom-made functional insole was more expensive (£656 vs. £554, *p*<0.001) to provide than the prefabricated insole (Table 4).

**Table 2:** **Outcomes and between group comparisons across time for peak pressure**, **forefoot pressure time integral**, **total contact area and forefoot rate of loading**

**Outcome**	**Custom**-**made functional**						**Prefabricated**						Overall significance*
	**Baseline**			**6**-**months**			**Baseline**			**6**-**months**			(**all randomised**)
	**Actual**	**%**	**95% CI**	**Actual**	**%**	**95% CI**	**Actual**	**%**	**95% CI**	**Actual**	**%**	**95% CI**	
**Peak pressure reduction kPa**	189	37	40, 33	239	37	42, 33	199	35	39, 31	187	31	34, 26	*F*=2.687
	(111)	(15)		(171)	(14)		(115)	(15)		(141)	(17)		Eta^2^=0.023
													*p*=0.104
**Forefoot pressure time integral reduction kPa**.**sec**	14	27	29, 24	18	30	32, 27	12	22	25, 20	15	24	26, 21	*F*=11.276
	(10)	(11)		(12)	(10)		(7)	(8)		(12)	(10)		Eta^2^=0.089
													*p*=0.001
**Total contact area increase mm**^**2**^	4285	32	30, 35	2443	15	13, 16	3827	29	27, 32	2395	15	14, 16	*F*=1.66
	(1464)	(10)		(1147)	(5)		(1183)	(9)		(803)	(5)		Eta^2^=0.014
													*p*=0.20
**Forefoot rate of loading reduction KPa**/**sec**	4	3	6, 0.4	7	4	8, 0.6	7	6	9, 3	6	3	6, 0.7	*F*=0.225
	(11)	(13)		(16)	(14)		(13)	(11)		(17)	(13)		Eta^2^=0.002
													*p*=0.636

Values are mean and (SD).

% is the actual difference expressed as a percentage of ‘no-insole’.

*ANOVA; significance of difference in % change between custom-made functional and prefabricated insoles over 6 months.

**Table 3:** Outcomes and between group comparisons across time for Bristol Foot Score and Audit of Diabetes Dependent Quality of Life

**Outcome**	**Custom**-**made functional** (**n**=**60**)				**Prefabricated** (**n**=**59**)				Overall significance*
	**Baseline**	**95% CI**	**6 month**	**95% CI**	**Baseline**	**95% CI**	**6 month**	**95% CI**	**(all randomised)**
**Bristol Foot Score**	44	41, 47	41	39, 44	42	39, 45	40	37, 43	*F*=0.449
	(13)		(12)		(12)		(11)		Eta^2^=0.004
									*p*=0.481
**Audit of Diabetes Dependent Quality of Life**	−2.2	−2.7, -1.7	−2.54	−3.1, -1.9	−2.39	−2.8, -1.9	−2.39	−3.0, -1.8	*F*=0.002
	(1.8)		(2.27)		(2.06)		(2.5)		Eta^2^=0.000
									*p*=0.963

Values are mean and (SD).

*ANOVA; significance of difference in patient-based measures between custom-made functional and prefabricated insoles over 6 months.

**Table 4:** Comparison of costs between the two insoles

**Outcome Mean Cost (£)**	**Custom**-**made functional**	**Prefabricated**	Overall significance*
**Insole**	137.65	31.73	(Fixed price)
**Clinician time**	158.28 (49.55)	163.59 (40.75)	*t*=0.638, *p*=0.525
**Patient time**	16.95 (5.31)	17.53 (4.36)	*t*=0.649, *p*=0.517
**Patient travel**	43.15 (22.39)	41.44 (16.72)	*t*=−0.470, *p*=0.639
**Total**	656.03 (69.56)	554.28 (53.34)	*t*=−8.942, *p*<0.001

Values are mean cost (£) and (SD).

*Independent sample t-tests; significance of difference between custom-made functional and prefabricated insole groups.

### Blind testing

To assess for bias and breaking the blinding process, participants were asked at completion of the study to guess their intervention group assignment. Of the 45 respondents receiving the prefabricated insole, 25 (56%) thought they had been given the custom-made functional insole, 4 (8%) thought they had the prefabricated insole and 16 (36%) did not know. Of the 46 respondents receiving the custom-made functional insole, 30 (65%) thought they had been given the custom-made insole, 4 (9%) thought they had been given the pre-fabricated insole and 12 (26%) did not know. A total of 34 (37%) participants chose the intervention received, 29 (32%) guessed incorrectly and 28 (31%) were unable to decide which insole they had been provided. Participants remained blind to the intervention allocation for the trial duration.

### Adverse events

Five (4%) of the 119 recruited participants developed adverse effects whilst participating in the trial. Four of the five had been randomly allocated the prefabricated insole: one developed a Charcot joint and the other three plantar ulcers, one of which was apparently caused by a sock being inadvertently left in the toe of a shoe while worn. One further participant that experienced an adverse event had received the custom-made insole: at follow-up, a 1^st^ interphalangeal joint ulcer was discovered in conjunction with suspected osteomyelitis, so the participant was immediately withdrawn from the study to be managed appropriately.

## Discussion

Both insoles were comparable in reducing peak pressure (for all foot types) and rate of forefoot loading whilst increasing total contact area, but the custom-made functional insoles were more expensive than the prefabricated insoles. Peak pressure is the measure that has been traditionally used in studies evaluating the effect of offloading. However, in addition, we used four other measures. For one of these, forefoot pressure time integral, the custom-made insoles performed significantly better, and these differences were maintained over the six month study period.

Total peak pressure was selected as the traditional measure of the effect of footwear and insole efficacy to ease comparison of results between studies. Forefoot pressure time integral was selected to reflect the increased risk of ulceration over the forefoot. More recently it has been reported that peak pressure and pressure time integral are inter-dependent and that within clinical trials significant differences in patterns found between the two parameters are generally minimal [20,21]. Thus, there is little value in routinely reporting both measures [20,21]. We acknowledge, therefore, that forefoot peak pressure would have been an equally suitable measure and accept that our findings are likely to have been the same if forefoot peak pressure had been selected [20,21].

No other randomised controlled trial has been identified to indicate that custom-made functional insoles are significantly more effective in reducing forefoot pressure time integral or forefoot peak pressure than prefabricated insoles when used to reduce ulcer risk in neuropathic diabetic feet. Reductions in peak pressure observed at insole issue in our study (31-37%) are, however, comparable to peak pressure reductions demonstrated by other non-randomised studies (32%) [22,23]. At six month follow-up, peak pressure reductions observed in our trial (Prefabricated 31%, Custom-made functional 37%) appear better than findings presented elsewhere. Mohamed and colleagues [22] compared a custom plastazote orthosis to a custom aliplast/plastazote orthosis in two groups of eight people with diabetic neuropathy and reported peak pressure reduction of 26% at three months. Lobman and colleagues [23] compared 18 participants with neuropathy provided with custom made EVA insoles in therapeutic shoes with 63 controls and reported a mean reduction in peak pressure of 28% at six months. The apparent enhanced performance across time of the insoles evaluated by our trial may reflect the greater durability of materials selected. Mohamed and colleagues [22] describe making modifications to the plastazote insoles after only one month of use to compensate for material compression.

Reduction in peak plantar pressure is believed to result from a corresponding increase in total surface area. We found that although both insoles increased total contact area significantly, that change had reduced by 50% at six months follow-up. If peak pressure was strongly associated with total contact area, a reduction in total contact area would generate an associated increase in peak pressure. Conversely, the results of our study found that at six months follow-up, whilst the effect of the insoles on total contact area had decreased, the effect on peak pressure stayed constant.

The absence of association between peak pressure and total contact area in this trial may be explained by considering the dynamic nature of the data. Most peak pressure sites were located over the forefoot during propulsion. In contrast, the insole generated the greatest increase in surface area beneath the medial longitudinal arch, at midstance. Thus, the effect of increased total contact area on peak pressure at midstance may not carry over into propulsion. The mechanism by which insoles reduce plantar peak pressure is unclear and in need of further investigation.

Two non-randomised studies confirm an increase in total contact area when insoles were worn [24,25]. Albert and Rinoie [26] recorded a 5-10% increase in total contact area when a rigid custom-made device was worn; this increase was constant over the 3-month study period. Raspovic and colleagues [24] used the F-scan® to evaluate change in contact area, testing a range of customised accommodative insoles in a sample of 8 high risk people with diabetic neuropathy and a history of ulceration. A 19% increase in total contact area was found when wearing the insole, no follow-up evaluation was undertaken. In comparison, our study reported a substantially greater total contact area increase of 32% and 29% for the custom-made functional and prefabricated insoles at issue, reducing to 15% at six months.

Differences between study results may be attributed to differences in methodology. Raspovic and colleagues [24] investigated the effect of previously worn non-casted custom-made insoles on total contact area. Compared to our study insoles, the profile of the non-casted device may be less contoured, thus reducing the comparative total surface area. Equally the 19% increase in total contact area reported by Raspovic and colleagues [24] may reflect the time from issue; their findings are comparable with the 15% increase reported by our study at six month follow up.

Albert and Rinoie [25] included only people with diabetes and pronated feet. The contact area beneath pronated feet is high, thus reducing the capacity for an insole to further increase total contact area. A greater effect therefore might be predicted in a sample population not limited by foot type, such as that recruited for our trial (44 out of 119 had pronated feet in our sample). Furthermore, unlike our semi-rigid insoles, the insole investigated by Albert and Rinoie [25] was of rigid construction, unlikely to flex under load. Differences in insole flexibility offer further explanation for differences in change in total contact area reported between studies.

The custom-made functional device fabricated for clinical use is commonly prescribed to include the biomechanical features best suited to foot type (pronated or supinated). Likewise, the custom-made functional device designed for this trial incorporated biomechanical features specific to foot type in conjunction with features considered safe for the insensate foot. More than one third of study participants randomised to the custom-made functional insole group (n=25/60) received an insole intended to benefit the pronated foot. Although direct comparison between studies is not possible, the 37% reduction in peak pressure achieved by the custom-made insole in this trial appears similar to the clinical benefit achieved by the rigid device evaluated by Albert and Rinoie.

The outcome measure, duration of load as a percentage of stance at the site of peak pressure was included in recognition of the contribution toward ulceration that increased load times are thought to play [26,27]. No other study considering this measurement was found in the literature. The effect of wearing insoles on duration of load as a percentage of stance was variable and inconsistent between participants and when the same participant was measured on different occasions. Duration of load as a percentage of stance over the site of peak pressure cannot easily be altered by insole therapy and does not appear a suitable measurement to assess the effect of insoles in people with diabetes and neuropathy. However, given the limited research using this outcome measure, further work is required to determine whether these findings are representative of a general trend before a final conclusion can be drawn.

This study found that wearing insoles reduced rate of forefoot loading in the diabetic neuropathic foot, although the effect was small. It has been suggested that rate of forefoot loading maybe as important as the magnitude of load in ulcer formation [28], although an association with ulceration has not been determined. The only other study that we have found that assessed change in rate of loading in the evaluation of insoles for the management of the neuropathic foot [24], was non-randomised and recruited only eight participants so was likely to be under-powered.

Neither the ADDQoL nor the Bristol Foot Score showed any difference between the two insoles. Compared to other populations with diabetes, this sample of 119 participants with diabetic peripheral neuropathy had worse health-related quality of life. A survey of 795 outpatients attending annual review at a UK hospital diabetes clinic reported mean weighted impact score for health-related quality of life of −1.98, which compares to a mean weighted score for participants in this trial of −2.38 [29]. No other study has employed the ADDQoL to capture the impact of diabetic peripheral neuropathy on health-related quality of life.

The estimated direct cost of treating a diabetic ulcer over a 12 year period is £27,000 [30]. Assuming insoles are replaced annually and footwear two yearly, the estimated cost of insole provision over a 12 year period was calculated at £4771 and £3500 for the custom-made functional and prefabricated insole respectively. Therefore, each diabetic foot ulcer prevented by insole provision offers a potential cost saving of approximately £23,000. Full utility analysis using longitudinal measures of time to foot ulceration or life expectancy is needed to provide robust economic analysis of insoles for diabetic neuropathic foot ulceration.

There is no clear evidence that the custom-made functional or prefabricated insole is best practice for all diabetic neuropathic feet, however both are of value for reducing ulcer risk in people with diabetes and neuropathy. Both custom-made functional and prefabricated insoles were equally effective in reducing peak pressure; therefore the less expensive prefabricated insole is likely to be most cost effective. Practitioners tasked with accessing the diabetic foot for insole provision should, where appropriate, consider prescribing the more cost effective prefabricated insole. The custom-made functional insole was found slightly more effective than its cheaper counterpart in reducing forefoot pressure time integral. The clinical significance of reducing forefoot pressure remains undetermined. Further research is needed to determine; (i) which parameter is more important in predicting neuropathic foot ulceration, (ii) the magnitude of reduction deemed clinically sufficient to produce a symptom change, and (iii) confirmation of costs over time.

Of the 104 participants completing our study, 42 (40%) reported full compliance equating to wearing the insoles and shoes for a minimum of 7 hours a day, 7 days per week for a period of six months [31]. Chantelau and Haage [31] reported that participants with diabetic neuropathy and a history of foot ulceration, wearing protective shoes for >60% of the daytime reduced ulcer relapse rate by 50%. Thus, for the purposes of this study, 60% of daytime wear of insoles and footwear for the duration of the six month study were considered compliant. Footwear compliance within this trial, although apparently low, is favourable compared to compliance rates reported elsewhere [31,32]. Three participants did not wear the insoles because they felt unstable when walking with them. However, the most common reason given by participants for not wearing the insoles and shoes for more hours per day, was that they were being removed whilst indoors, despite footwear education to the contrary. Improving insole and footwear compliance in patients presenting with diabetic neuropathy is crucial to the success of diabetic foot health maintenance. Insole compliance may be improved if patients were provided with both indoor and outdoor footwear to accommodate insoles.

This study needs to be considered in light of a few limitations. Firstly, although all the tests were pre-specified, by exploring four outcomes, in addition to peak pressure, we increased the possibility of finding a significant result by chance. However, even adjusting the *p*-value for multiple testing, the difference in pressure time-integral is significant. Secondly, the clinical environment we used to collect data may not fully replicate the day-to-day conditions within which the insoles are required to function. The choice of footwear used to accommodate insoles can affect function. Therefore, care must be taken not to generalise the findings of this trial beyond the type of therapeutic footwear provided within the study. The six month follow up in our trial gave limited information regarding insole durability. In practice, frequency of insole replacement often extends beyond six months and is usually determined by physical signs of insole wear or changes in foot health. Finally, application to clinical practice may be enhanced if future studies were specific not only to foot pathology but foot type (pronated or supinated). The implication that forefoot pressure time integral and other selected variables are symbolic of ulceration risk is too simplistic and should be approached with caution; the aetiology of diabetic neuropathic ulceration is multi-factorial and complex, therefore whilst clearly relevant to ulceration risk, kinetic parameters are merely surrogate measures of internal tissue stress. Moreover, the actual pressure threshold generating internal tissue stress above which ulceration is inevitable is undetermined, therefore estimates of clinical significance are difficult to predict. Further studies are required using a randomised controlled trial design to assess insoles used for the prevention of diabetic neuropathic foot ulceration, particular attention should be given to the comparison of insole type using incidence of ulceration as a primary measure of outcome.

## Conclusion

This randomised controlled trial is novel in its comparison of insoles designed specifically to address the neuropathic foot-related biomechanical complications associated with ulceration development. Moreover, it is one of very few randomised controlled trials to control for footwear. The relevance to clinical practice is enhanced by the recruitment of a sample population including patients presenting with diabetic peripheral neuropathy but excluding peripheral arterial disease. The findings provide further evidence to suggest that insoles are of value and should form part of the plantar load reducing strategy for the diabetic neuropathic foot. Custom-made insoles are more expensive than prefabricated insoles and from our findings, no better in reducing risk. We recommend that practitioners tasked with accessing the diabetic foot for insole provision should, where appropriate, consider prescribing the more cost effective prefabricated insole. Insole and footwear compliance remains an issue and must be improved to enable the diabetic neuropathic population to fully benefit from any treatment effect.

## Competing interest

The authors declare that they have no competing interest.

## Authors’ contributions

JP conceived and conducted the trial, extracted and analysed the data and produced the initial draft manuscript. RJ, ES and GB critically reviewed the design, development and progress of the trial. RJ and ES reviewed the manuscript for academic content and GB reviewed the discipline specific content. DZ helped with the re-analysis of data dealing with reviewers’ concerns on initial submission. All authors read and approved the final manuscript.

## Supplementary Material

Additional file 1 **Summary of prescription writing protocol and rationale[**[33-52]**].** Click here for file
