# Supplementary material for: A comparison of customised and prefabricated insoles to reduce risk factors for neuropathic diabetic foot ulceration: a participant-blinded randomised controlled trial
Source: J Foot Ankle Res. 2012 Dec 5;5:31. doi: 10.1186/1757-1146-5-31 (PMC3554426; doi:10.1186/1757-1146-5-31)
Supplement: Additional file 1 — Summary of prescription writing protocol and rationale[[33-52]]. [file 1757-1146-5-31-S1.doc]

Additional File 1: Summary of prescription writing protocol and rational

| **Key finding from the literature** | **Clinical implication** | **Insole design objective** | **Insole modification** |
| --- | --- | --- | --- |
| Foot structure is predictive of plantar pressure [1-3] | Consider individual variation in foot structure with influence over magnitude and location of peak plantar pressure | The profile of the insole must reflect foot type and function to;   1. Increase contact area 2. Modify abnormal loading patterns | - Foot posture index < +4 take a weight-bearing impression of the foot - Foot posture index ≥+4 take a semi-weight bearing impression of the foot with subtalar joint (STJ) held in neutral - In the presence of a medially deviated STJ axis incorporate a 4mm medial hell skive |
| Individuals with diabetes display reduced joint range of motion [4-7] | Minimise the required STJ range of motion for dynamic function. Closer align available and required joint range of motion. | Support and/or accommodate forefoot deformity reducing the need for STJ compensation motion | - In the presence of a plantarflexed 1st ray incorporate a cut-out under the 1st metatarsophalangeal joint - Foot Posture Index FPI ≥ +4 take a semi weight-bearing impression of the foot with STJ neutral maintained. Apply dorsal pressure to the medial forefoot to reduce any forefoot supinatus present and achieve a fully plantar-grade cast |
| Individuals with diabetes display reduced joint range of motion [4-7] | Minimise excessive transverse plane motion between foot and supporting surface | The contact profile of the insole will silhouette the profile of the individual foot to obtain a secure, precise fit | - The each insole must be custom manufactured from a accurate impression of the individual foot |

**Table 1: Summary of prescription writing protocol**

**Rational for prescription writing protocol**

This prescription writing protocol uses the principles of biomechanics to relate relevant research evidence to the findings of a biomechanical examination in order to suggest the possible design implications for insoles to reduce ulcer risk in the diabetic neuropathic foot. The protocol provides a simple, clinically useful, evidence based, custom-made functional insole protocol specific to the neuropathic diabetic foot. Our RCT investigated the application of the custom-made functional insole design protocol in the preventative management of people with diabetes and neuropathy. The study compared the affects of two types of insole 1) a custom-made functional device prescribed according to the custom-made functional insole design protocol and 2) a prefabricated device.

Studies investigating a correlation between foot structure and plantar pressure generally employ X-ray or similar imaging method to record detailed, quantifiable variants in foot anatomy [1-3]. This method of structural analysis is often not readily available, thus not appropriate for routine foot assessment within current clinical practice. Therefore an alternative clinical measure of foot structure was sourced for use within the custom-made functional insole prescription writing protocol. Validated, quick to perform and low cost, the foot posture index offered a suitable clinical method of ranking foot structure [8-10]. The method enabled foot structure to be ranked according to degree of pronation or supination evident in relaxed bi-pedal stance [8] and could be integrated within the custom-made functional insole prescription writing protocol.

Several studies correlating foot structure and pressure conclude that first metatarsal shaft and calcaneal inclination angles are important predictors of plantar pressure [1, 2]. Both variables can be considered surrogate measures of medial longitudinal arch height, suggesting that highly arched feet are associated with increased localised plantar pressure. The contact surface area of the highly arched foot tends to be less than that of the low arched foot. Contact surface area is believed to be inversely proportional to pressure; therefore increasing surface area in the neuropathic diabetic foot may reduce plantar pressure and ulceration risk [11, 12]. Thus highly arched, supinated feet scoring a foot posture index of <+4 received an insole designed to maximise total contact area. This was achieved by taking a weight-bearing cast of the foot to maximise surface contact area between plantar foot and insole whilst reducing the anti-pronatory action of the insole.

The insole prescription writing protocol assumed peak pressure in pronated neuropathic diabetic feet (FPI≥+4) was located medial to the plantar forefoot. This hypothesis is supported by published work correlating pronated diabetic foot type with medial ulceration site [13, 14]. The protocol recommended that when foot posture index ≥ +4, a semi-weight bearing impression should be taken of the foot. This impression technique is designed to raise the medial long arch of the insole, increase the supination moments medial to the subtalar joint axis and re-locate peak forefoot pressure laterally.

Within this RCT a medially deviated subtalar joint axis was defined using the method described by Kirby [15]. In a foot displaying a medially deviated subtalar joint axis it is possible for ground reaction force applied medial to the long arch to act lateral to the subtalar joint axis, consequently generating an undesirable pronation moment [16-18]. To combat this anomaly the medial heel skive insole modification has been developed [19]. This prescription modification amplifies ground reaction force underlying the medial heel region; an area in the large majority of feet situated medial to the STJ axis, thus assuring the generation of intended supination moments on weight bearing [19]. In the presence of a medially deviated subtalar joint axis the custom-made functional prescription writing protocol recommended the addition of a medial heel skive.

In most feet a biomechanical forefoot deformity will impose demands for compensation motion from adjacent joints altering the dynamic position of the foot during weight-bearing activity [20]. For example, an inverted forefoot deformity will encourage compensatory subtalar and mid-tarsal joint pronation to occur in response to increased ground reaction force lateral to the subtalar joint axis when weight bearing [21]. The subtalar joint pronates until equilibrium is reached; that is the supination moment about the joint axis is equal to the pronation moment, usually when the forefoot rests plantar-grade to the ground. Conversely a plantarflexed 1^st^ ray promotes compensatory mid-tarsal and secondarily subtalar joint supination in response to increased ground reaction force medial to the subtalar joint axis [20].

Limitation in subtalar and mid-tarsal joint motion is common in the feet of people with diabetes and previous neuropathic ulceration [6, 14]. If the available range of subtalar and mid-tarsal joint motion is limited and the ability of the foot to provide compensatory frontal plan motion will be compromised. Abnormal but inadequate subtalar and mid-tarsal joint compensation in response to forefoot biomechanical deformities may lead to uneven distribution of forefoot load, focal areas of increased forefoot pressure and ulceration [13, 22].

The biomechanical objective of the insole may be to reduce demand for frontal plane compensation motion, to maximise the functional efficiency of the available limited range of motion. In the presence of a plantarflexed 1st ray a cut-out underlying the 1^st^ MTP was incorporated within the insole during fabrication. The 1^st^ MTP cut-out accommodates the prominent 1^st^ MTP joint redistributing forefoot load more evenly and removing the compensation requirement.

In the presence of a forefoot supinatus deformity with a foot posture index ≥+4, a semi-weight-bearing foot impression was taken with the supinatus deformity reduced by manipulation; forefoot supinatus deformity is believed to be a flexible forefoot deformity [20]. The effect was to increase medial long arch height of the finished insole and potentially reduce and correct the forefoot deformity over time.

During normal daily activities the lower limb undergoes transverse plane rotation. This transverse plane rotation is largely redirected proximally to the plantar foot by way of subtalar joint linkage between lower limb and foot, expressed as talar adduction and abduction with closed chain pronation and supination [21]. Limitation in available subtalar joint motion to a level below that required to absorb transverse lower limb rotation may induce abnormal and excessive transverse plane motion of the foot within shoe. This unwanted motion, manifested as potentially destructive shear stress within plantar tissues, may be limited by the closely fitting confines of an insole shaped to retain the foot in the transverse plane. Therefore each insole was custom manufactured to replicate the contours of the foot using CAD/CAM technology from an accurate impression of the individual foot.

**References**

[1] Cavanagh P, Morag E, Boulton A, Young M, Deffner K, Pammer S: **The relationship of static foot structure to dynamic foot function**. *Journal of Biomechanics.* 1997, **30** (3):243-50.

[2] Morag E, Cavanagh P: **Structural and functional predictors of regional peak pressures under the foot during walking**. *Journal of Biomechanics.* 1999, **32**:359-70.

[3] Mueller M, Hastings M, Commean P, Smith K, Pilgram T, Robertson D*, et al.*: **Forefoot structural predictors of plantar pressures during walking in people with diabetes and peripheral neuropathy**. *Journal of Biomechanics.* 2003, **36** (7):1009-17.

[4] Glasoe W, Allen M, Ludewig P, Saltzman C: **Dorsal mobility and first ray stiffness in patients with diabetes mellitus**. *Foot and Ankle International.* 2004, **25** (8):550-5.

[5] Fernando D, Masson E, Veves A, Boulton A: **Relationship of limited joint mobility to abnormal foot pressures and diabetic foot ulceration**. *Diabetes Care.* 1991, **14** (1):8-11.

[6] Delbridge I, Perry P, Marr S, Arnold N, Yue D, Turtle J*, et al.*: **Limited joint mobility in the diabetic foot: relationship to neuropathic ulceration**. *Diabetic Medicine.* 1988, **5**:333-7.

[7] Burton J: **Thick skin and stiff joints in insulin-dependent diabetes mellitus**. *British Journal of Dermatology.* 1982, **106**:369-71.

[8] Evans AM, Copper AW, Scharfbillig RW, Scutter SD, Williams MT: **Reliability of the foot posture index and traditional measures of foot position.** *Journal of the American Podiatric Medical Association.* 2003, **93** (3):203-13.

[9] Keenan A-M, Redmond AC, Horton M, Conaghan PG, Tennant A: **The Foot Posture Index: Rasch analysis of a novel, foot-specific outcome measure.** *Arch Phys Med Rehabil.* 2007, **88** (1):88-93.

[10] Scharfbillig R, Evans AM, Copper AW, Williams M, Scutter S, Iasiello H*, et al.*: **Criterion validation of four criteria of the foot posture index.** *Journal of the American Podiatric Medical Association.* 2004, **94** (1):31-8.

[11] Bus S, Ulbrecht J, Cavanagh P: **Pressure relief and load redistribution by custom-made insoles in diabetic patients with neuropathy and foot deformity**. *Clinical Biomechanics.* 2004, **19** (6):629-38.

[12] Kato H, Takada T, Kawamura T, Torii NHS: **The reduction and redistribution of plantar pressures using foot orthoses in diabetic patients**. *Diabetes Research in Clinical Practice.* 1996, **31**:115-8.

[13] Bevans J: **Biomechanics and plantar ulcers in diabetes**. *The Foot.* 1992, **2**:166-72.

[14] Mueller M, Diamond J, Delitto A, Sinacore D: **Insensitivity limited joint mobility and plantar ulcers in patients with diabetes mellitus**. *Physical Therapy.* 1989, **69** (6):453-62.

[15] Kirby KA: **Methods for determination of positional variations in the subtalar joint axis.** *Journal of the American Podiatric medical Association.* 1987, **77** (5):228-34.

[16] Kirby KA: **Subtalar joint axis location and rotational equilibrium theory of foot function.** *Journal of the American Podiatric Medical Association.* 2001, **91** (9):465-87.

[17] Kirby KA: **Biomechanics of the normal and abnormal foot.** *Journal of the American Podiatric Medical Association.* 2000, **90** (1):30-4.

[18] Kirby KA: **Rotational equilibrium across the subtalar joint axis.** *Journal of the American Podiatric Medical Association.* 1989, **79** (1):1-14.

[19] Kirby KA: **The medial heel skive technique. Improving pronation control in foot orthoses.** *Journal of the American Podiatric Medical Association.* 1992, **82** (4):177-88.

[20] Root M, Orien W, Weed J, eds. Clinical Biomechanics Volume II. Normal and abnormal function of the foot: Los Angeles, California, USA 1977.

[21] Valmassy R: **Clinical Biomechanics of the Lower Extremity**. St Louis: Mosby 1996.

[22] Mueller M, Minor S, Diamond J, Blair III V: **Relationship of foot deformity to ulcer location in patients with diabetes mellitus**. *Physical Therapy.* 1990, **70** (6):356-62.
